# Supplementary material for: Evidence supported by Mendelian randomization: impact on inflammatory factors in knee osteoarthritis
Source: Front Med (Lausanne). 2024 May 28;11:1382836. doi: 10.3389/fmed.2024.1382836 (PMC11165061; doi:10.3389/fmed.2024.1382836)
Supplement: Supplementary file 1 [file Data_Sheet_1.ZIP › data/GCST90274822_5e6/822.forest.pdf]

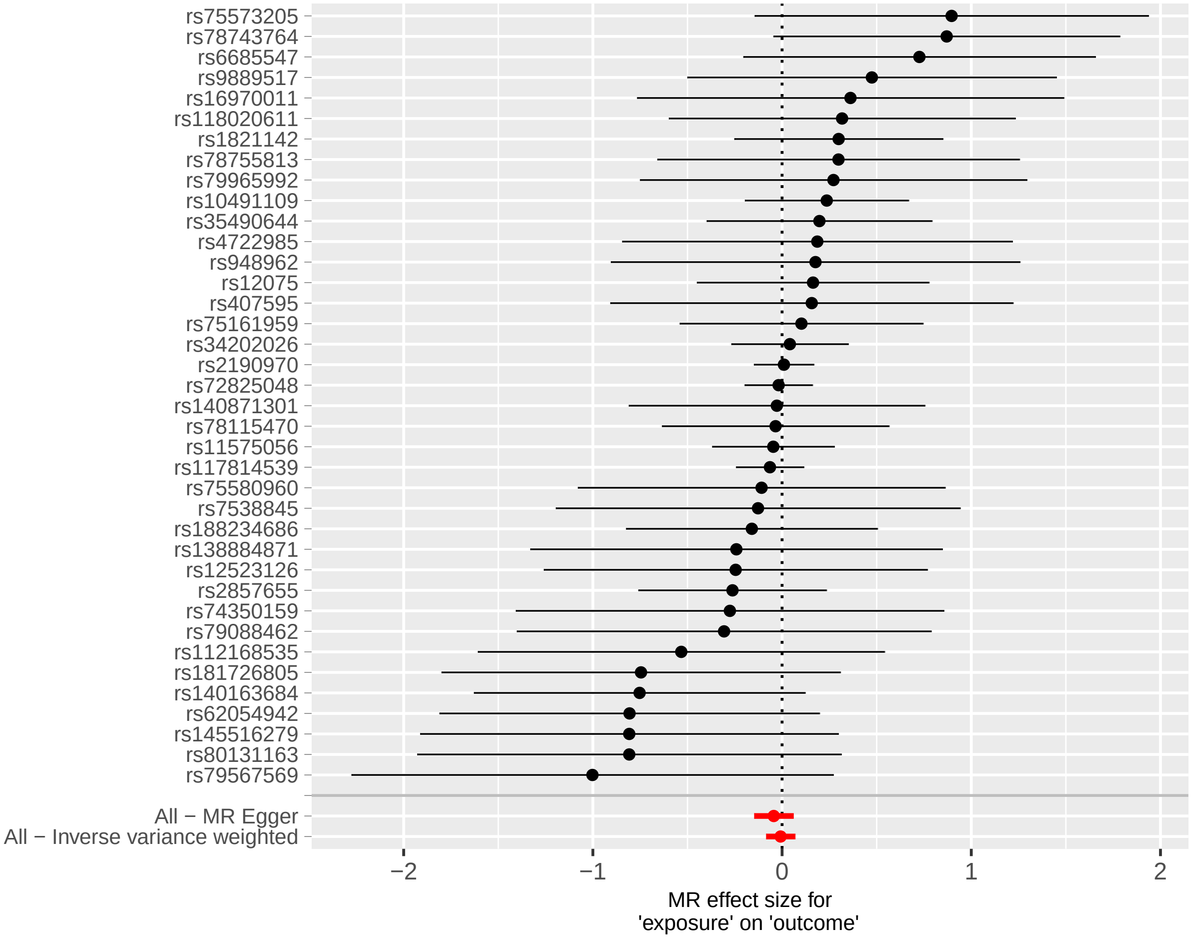
rs75573205
rs78743764
rs6685547
rs9889517
rs16970011
rs118020611
rs1821142
rs78755813
rs79965992
rs10491109
rs35490644
rs4722985
rs948962
rs12075
rs407595
rs75161959
rs34202026
rs2190970
rs72825048
rs140871301
rs78115470
rs11575056
rs117814539
rs75580960
rs7538845
rs188234686
rs138884871
rs12523126
rs2857655
rs74350159
rs79088462
rs112168535
rs181726805
rs140163684
rs62054942
rs145516279
rs80131163
rs79567569
All − MR Egger
All − Inverse variance weighted
−2
−1
0
1
2
MR effect size for
'exposure' on 'outcome'
